# From the Amazon: A comprehensive liver transcriptome dataset of the teleost fish tambaqui, *Colossoma macropomum*

**DOI:** 10.1016/j.dib.2019.103751

**Published:** 2019-03-07

**Authors:** André M. Machado, Renato Ferraz, Ricardo do Amaral Ribeiro, Rodrigo Ozório, L. Filipe C. Castro

**Affiliations:** a CIIMAR – Interdisciplinary Centre of Marine and Environmental Research, U. Porto – University of Porto, Porto, Portugal; b ICBAS – Institute of Biomedical Sciences Abel Salazar, U. Porto – University of Porto, Portugal; c Universidade Federal Do Acre, Brazil; d Department of Biology, Faculty of Sciences, U. Porto – University of Porto, Portugal

**Keywords:** Tambaqui, Transcriptome, Liver, Aquaculture

## Abstract

The teleost fish tambaqui, *Colossoma macropomum*, is a valuable resource for the Brazillian aquaculture sector, representing more than one-quarter of the total production. In this context, the development of molecular tools is paramount to address and improve productivity, nutrition, and genetic breeding programs. In this study, we applied RNA-seq technology to produce the first comprehensive liver transcriptome in this species. Our analysis generated a *gold* standard transcriptome with a total of 43,098 transcripts, with an N50 of 1855 bp and the average length of 1312 bp. To functionally annotate the transcripts, the Trinotate pipeline together with several public databases were scrutinized. The blast-x analysis revealed more than 40,000 homologous match hits for each database (NCBI-Nr, Uniref90, Swissprot, Trembl), while the Kaas web server allowed the mapping of our transcripts to 380 kegg pathways. The dataset provided in this study entails a comprehensive molecular resource, which will be instrumental to further develop tambaqui aquaculture, specifically in the field of nutrigenomics.

**Table:** Specifications table

Subject area	Biology
More specific subject area	Transcriptomics
Type of data	RNA sequencing data
How data was acquired	Illumina HiSeq 4000 system
Data format	Raw data in FASTQ, filtered and gold standard transcriptome assembly FASTA format
Experimental factors	The liver sample was collected from one juvenile *Colossoma macropomum* specimen acquired at a commercial farm
Experimental features	De novo assembly of the transcriptome, functional annotation and general transcriptomic analyses
Data source location	Brazil (10.086483 S 67.537708 W)
Data accessibility	Raw FASTQ files can be found in NCBI SRA database. (https://www.ncbi.nlm.nih.gov/sra/SRX3714434). The filtered de novo transcriptome assembly was deposited in the NCBI TSA. (https://www.ncbi.nlm.nih.gov/nuccore/GGHL00000000.1). The gold Standard transcriptome is available in the figshare digital repository (https://figshare.com/s/d52c6698e6196bb6c8a5)
**Related research article:**	[1]

**Table:** 

**Value of the data** • Transcriptomic analyses provide important resources for multiple fields, such as to improve aquaculture production. • The teleost fish tambaqui (Colossoma macropomum) is a valuable resource for the Brazillian economy. • This data article reports the first liver transcriptome of the teleost fish tambaqui. • The present dataset will provide a good reference for future studies namely those associated with the nutritional requirements of this species*.*

## 1 Data

**Fig. 1:**
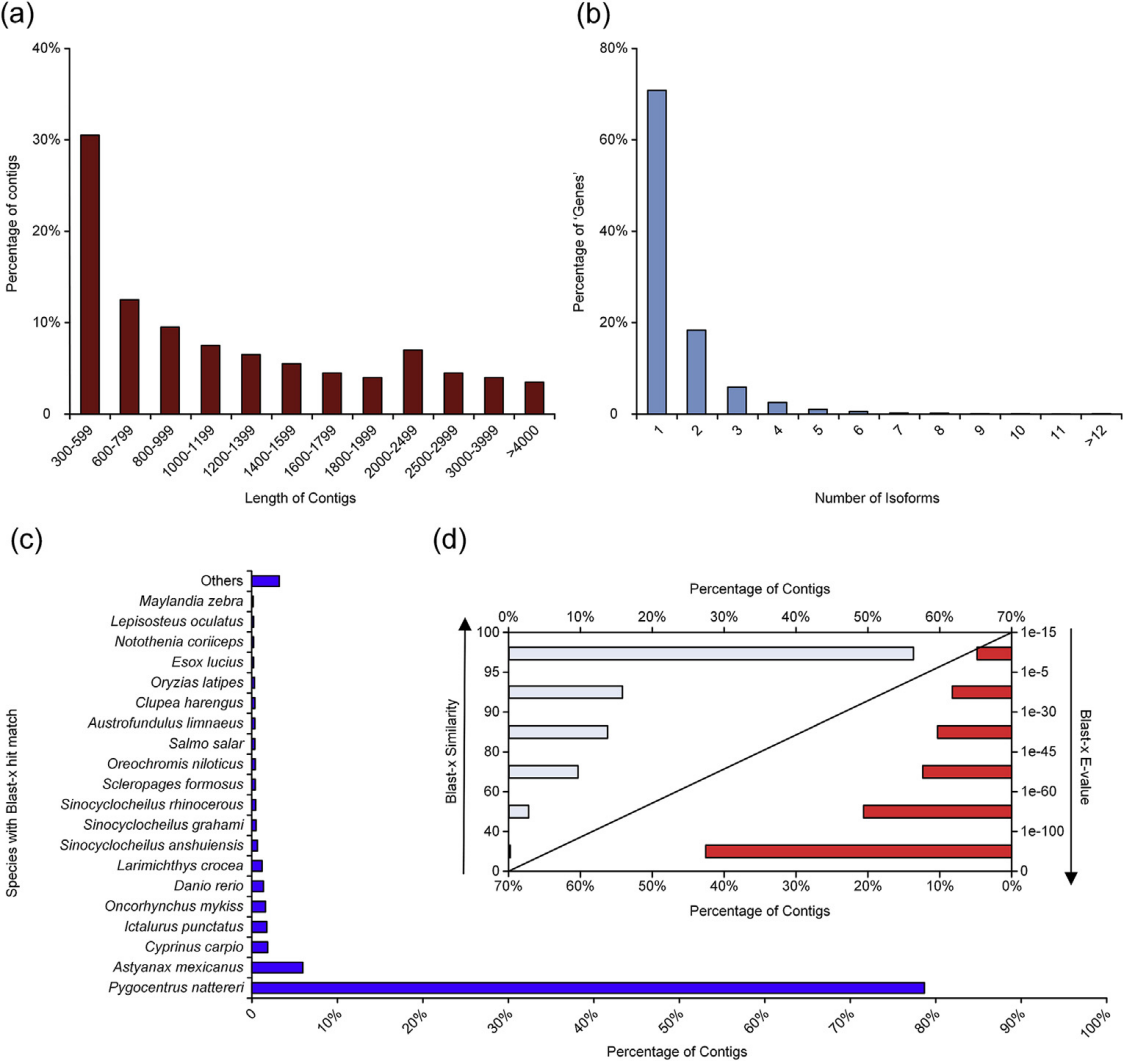
Quality assessment and blast-x analysis of the gold standard transcriptome of *C. macropomum*. a) Transcript length distribution; b) A number of transcripts (isoforms) per gene; c) Homologous gene-species distribution; d) E-value and Similarity distribution.

**Fig. 2:**
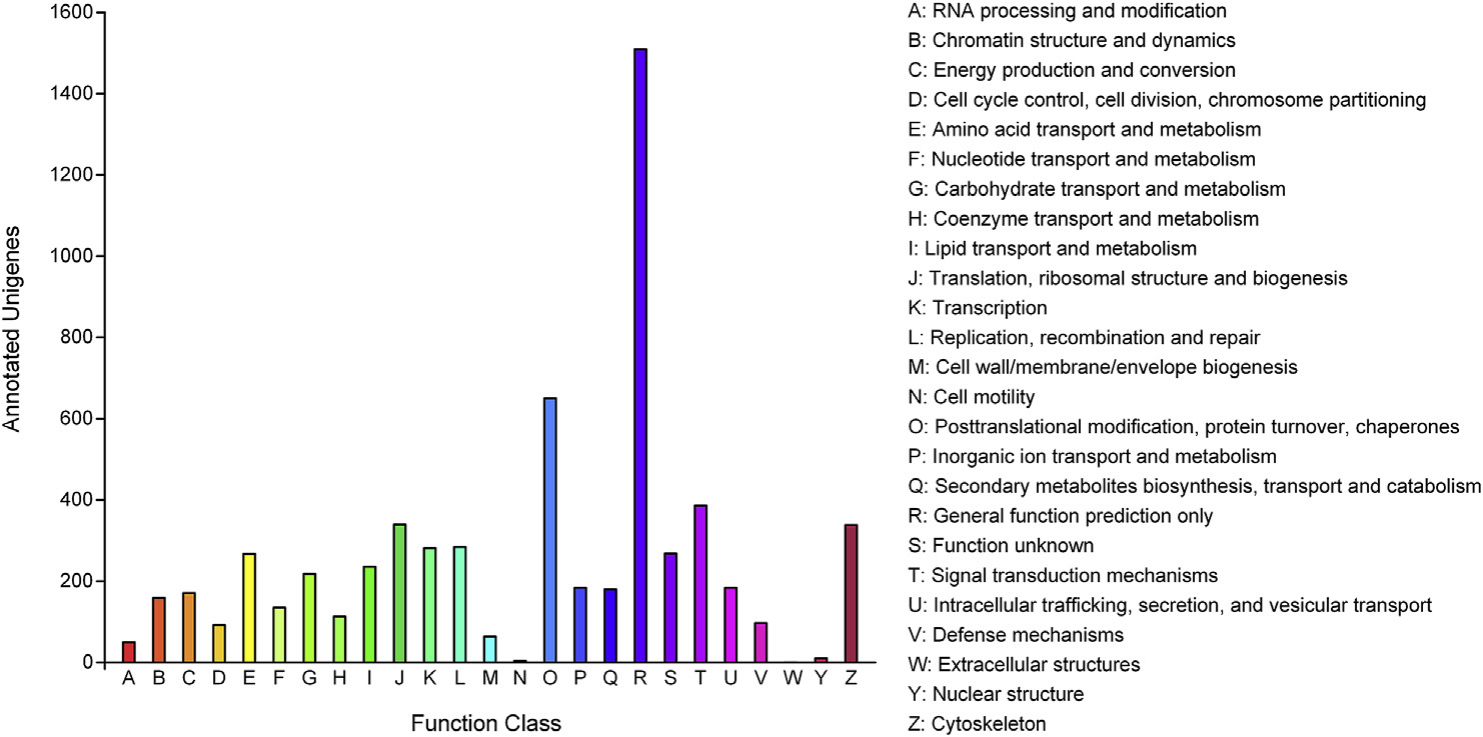
Histogram of the clusters of orthologous groups of *C. macropomum* transcriptome (COG).

**Fig. 3:**
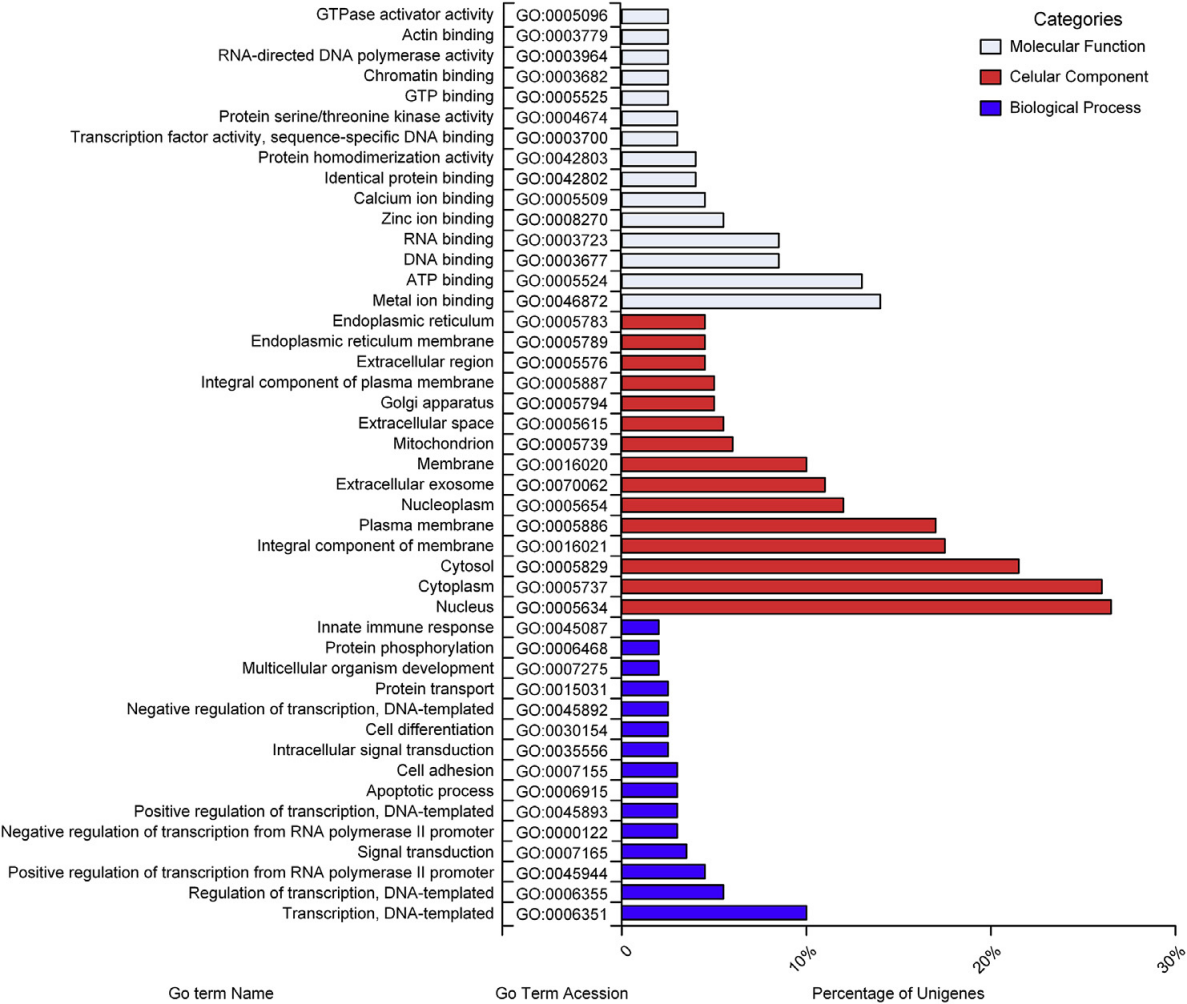
Functional classification of *C. macropomum* in three Gene Ontology (GO) categories-biological process (blue), molecular function (grey), cellular component (red).

This data article reports the first deep-sequencing of liver RNA of tambaqui (*Colossoma macropomum*, Cuvier,1818), which should prove valuable for investigations in other fields such as nutrigenomics [1]. We provide an extended molecular resource that integrates the assembly and annotation of the first liver transcriptome for this specie. In addition, we also deliver several analyses of Go terms, Kegg pathways and Clusters of Orthologous Groups focused on the general features of this dataset. Briefly: in Table 1 are described the *in silico* statistics of the original, filtered and gold standard transcriptome assembly; in Fig. 1e plotted the blast-x analysis of the gold standard transcriptome; in Fig. 2 it is possible to visualize the clusters of orthologous groups analysis; in Fig. 3 is displayed the Functional classification of tambaqui liver transcriptome into three Gene Ontology (GO) categories.

**Table 1:** Transrate and Trinity statistics of the original, filtered and gold standard transcriptome assembly of liver transcriptome of *C. macropomum.*

*C. macropomum* tissue	Liver		
Number of raw sequencing reads	52753287		
Number of cleaned reads used in the assembly	43509171		
Percentage of reads submitted to assembly (%)	82.48		
Assembly versions	Raw transcriptome assembly	Filtered de novo transcriptome assembly	Gold Standard transcriptome
Number of “genes”^a^	471503	30762	28638
Number of transcripts	659372	103895	43098
N50 transcript length (bp)^b^	1000	2517	1855
Median transcript length (bp)	466	1376	930
Mean transcript length (bp)	776	1811	1312
Length of the smallest transcript	301	301	301
Length of the largest transcript	19846	19846	18047
Number of transcripts with length > 1k nn^c^	122174	66468	20222
Number of transcripts with length > 10k nn^d^	285	282	36
Total Assembled bases	511615092	188186859	56527072

a Number of groups of transcripts clustered with base on shared sequence content.

b N50 is the sequence length of the shortest contig at 50% of the total transcriptome length.

c Number of transcripts with length larger than 1000 nucleotides.

d Number of transcripts with length larger than 10000 nucleotides.

## 2 Experimental design, materials and methods

### 2.1 Sampling, RNA isolation and RNA-Seq library preparation

Tambaqui juvenile specimens were obtained from a commercial farm in Rio Branco, Acre, Brazil (10°05′10.6″S 67°32′15.8″W) and transported to Universidade Federal do Acre, Brazil, where they were anaesthetized. Fresh liver was quickly collected and immediately preserved in an RNA stabilization buffer (RNAlater) and stored at −80 °C prior to RNA extraction. RNA extraction from the liver sample was performed with the kit (illustra RNAspin Mini RNA Isolation Kit, GE Healthcare, UK). The process included an on-column DNase I treatment (provided in the kit). RNA integrity was assessed on a 1% agarose TAE gel stained with GelRed™ nucleic acid stain (Biotium, Hayward, CA, USA). The generated high-quality liver total RNA sample was sequenced in the lllumina Hiseq 4000 platform, using 150bp paired-end sequencing reads by STABVIDA, Lda (Caparica, Portugal).

### 2.2 Cleaning, de novo assembly, and optimization of the liver transcriptome

The raw reads generated in sequencing were quality-checked with FastQC (https://www.bioinformatics.babraham.ac.uk/projects/fastqc/). Trimmomatic [2] was used to trim the first 15 bases of the reads and bases with a quality score below 15 at leading and trailing ends. Reads were then scanned with a 4-base sliding window, cutting when the average quality per base dropped below 20. In the end, only reads with high quality and longer than 50 bases were retained for further analysis. After applying a first technical quality control, the dataset was de novo assembled using the Trinity v2.5.0 software [3], following the Haas and co-workers protocol [4] and specific parameters to our case, strand-specific data and minimum length contig (SS_lib_type RF; min_contig_length 300). From this stage we obtained the Raw transcriptome assembly (Table 1).

The liver transcriptome optimization was done using three independent approaches. Firstly, the TransDecoder (https://transdecoder.github.io/) was used to predict open reading frames (ORFs) with a minimum cut-off of 100 amino acids, with recourse to homology searches (Blast-p against Swissprot Database [5] and Pfam search [6]) as auxiliary. Secondly, all transcripts were blasted against two independent databases, (NR) Non-Redundant of NCBI and Uniref90 of Uniprot [5]. To perform the blast, it was used the blast-x tool of DIAMOND v0.8.36 software [7] and all hits, given a match with *Actinopterygii* taxon with an E-value cut-off of 1e-5, were retained (Table 1). At this stage, the liver transcriptome filtration was done by overlapping the contigs with ORF to the match hits in *Actinopterygii* taxon (Filtered de novo transcriptome assembly).

Thirdly, the tr2aacds pipeline, from the Evidential – Gene package (http://arthropods.eugenes.org/EvidentialGene/), was used as a strategy to handle the redundancy and the number of isoforms per ‘gene’ in this filtered transcriptome. All transcripts classified by the tr2aacds pipeline as ‘primary’ or ‘alternate’ were retained to the next step of the annotation (Gold standard transcriptome assembly). To access the quality of the gold standard transcriptome, the distribution of sequence lengths and the number of isoforms per genes are shown in Fig. 1 a and b and Supplementary Table S1 and S2 in Supplementary File 1. Importantly, all steps of filtering and optimization were supervised with Trinity and Transrate [8] statistics (Table 1). Additionally, we also assessed the completeness of our transcriptome, in terms of gene content, using the eukaryota, and the metazoa lineage-specific profile libraries of Benchmarking Universal Single-Copy Orthologs tool (BUSCO) [9] (Supplementary Table S3 in Supplementary File 1).

### 2.3 Transcriptome annotation

To perform the transcriptome annotation, the final nucleotide and aminoacid sequences were retrieved from transdecoder.pep and Trinity.fasta initial files with the heads of the gold standard transcriptome assembly. Subsequently, the sequences were searched against several databases, NR, Uniref90, Trembl, Swissprot, at the local level, using blast-p and blast-x tools of the DIAMOND v0.8.36 software [7], and applying an E-value cut-off of 1e-5. To have a global overview of the orthologs genes contained in our transcriptome assembly, in relation to NR database of NCBI, the top 20 species distribution of blast-x against NR database, the similarity and e-value distributions are plotted in Fig. 1 c and d (Supplementary Tables S4, S5, S6 of Supplementary File 1). The PFAM [6] and HMMER [10] were used to identify protein domains, TMHMM [11] to predict transmembrane regions, GOseq [12] to determine GO and eggNOG v.3.0 [13] to identify clusters of orthologous groups of genes. All the results were integrated into the Trinotate v3.0.1 (http://trinotate.github.io) annotation pipeline and then reported with an E-value cut-off of 1e-5 (Supplementary Table S7 in Supplementary File 1).

### 2.4 Go terms, clusters of orthologous groups and Kegg patways analyses

The analysis of Go terms and COG's were done using the longest ORF per ‘gene’, unigenes, of the trinotate report (Annotation statistics to this dataset can be consulted under the sub-title of Final Transcriptome Subset, Supplementary Table S7 in Supplementary File 1). Regarding the search of COG's, it was done through the eggNOG database integrated within the Trinotate pipeline, and briefly, it was checked the percentage of unigenes annotated in at least one of the 25 COG categories contained in COG database. In Fig. 2 and in Supplementary Table S8 of Supplementary File 1 it is possible to access a graphical and tabular distributions of these analyses. The Go terms analyses were based on blast hits of SwissProt, and through this way we assign the unigenes to three main categories: molecular function, cellular component and biological process. The top 15 GO terms with a higher number of mapped unigenes were plotted in Fig. 3. In parallel, to improve the comprehension of functional and metabolic interactions in our transcriptome, the longest protein sequences, also were submitted to KAAS web server for KEGG annotation, using as reference 400,502 teleost fish sequences. This approach allowed to annotate and mapp a high number of unigenes to several metabolic patways (Supplementary Table S9 in Supplementary File 1).

## Ethics statement

The tissue sampling procedure was approved by the ethical committee of the Federal University of Acre, Brazil, with the project number 23107-009564/2014-29 and protocol number 08/201, under the responsibility of Prof. Anselmo Fortunato Ruiz Rodriguez. The samples were exported from Brazil to Portugal with the approved of the Brazilian Environmental and Renewable Resource Institute (IBAMA) with permit number 16BR021098/DF.
