# Supplementary material for: From the Amazon: A comprehensive liver transcriptome dataset of the teleost fish tambaqui, Colossoma macropomum
Source: Data Brief. 2019 Mar 7;23:103751. doi: 10.1016/j.dib.2019.103751 (PMC6660486; doi:10.1016/j.dib.2019.103751)
Supplement: Multimedia component 2 [file mmc2.docx]

Supplementary File 1

From the Amazon: a comprehensive liver transcriptome dataset of the teleost fish tambaqui, *Colossoma macropomum*

André M. Machado1+, Renato Ferraz1,2+, Mónica Lopes-Marques1, Ricardo do Amaral Ribeiro3, Rodrigo Ozório1, L. Filipe C. Castro1,4*

^1^CIIMAR – Interdisciplinary Centre of Marine and Environmental Research, U. Porto – University of Porto, Porto, Portugal

^2^ICBAS - Institute of Biomedical Sciences Abel Salazar, U. Porto - University of Porto, Portugal

^3^Universidade Federal do Acre, Brazil

^4^Department of Biology, Faculty of Sciences, U. Porto - University of Porto, Portugal

^+^These authors contributed equally to this work

^*^**Corresponding author:** [filipe.castro@ciimar.up.pt](mailto:filipe.castro@ciimar.up.pt)

**This Document Includes**:

**Supplementary Tables**

Supplementary Table S1. Length distribution of contigs in gold standard transcriptome assembly.

Supplementary Table S2. Distribution of isoforms per ‘gene’ in the gold standard transcriptome assembly.

Supplementary Table S3. BUSCO evaluations of completeness of C. macropomum Liver gold standard transcriptome against the metazoa and eukaryota gene sets.

Supplementary Table S4. The top 20 species for which there was a top Blast-x hit. These Blast-x results from the queried of gold standard transcriptome against Non-redundant Database of NCBI.

Supplementary Table S5. E-value distribution of Blast-x hits of gold standard transcriptome against NR database.

Supplementary Table S6. Similarity distribution of Blast-x hits of gold standard transcriptome against NR database.

Supplementary Table S7. Functional annotation categories and statistics for gold standard transcriptome and for unigenes.

Supplementary Table S8. Clusters of orthologous groups distribution.

**Supplementary Table S9.** Total number of KEGG pathways with the numbers of transcripts of tambaqui.

Supplementary Table S1. Length distribution of contigs in gold standard transcriptome assembly.

| **Contig Lenght** | **Number of Hits** | **Percentage of Hits (%)** |
| --- | --- | --- |
| 300-600 | 13,237 | 30.71 |
| 600-800 | 5,467 | 12.68 |
| 800-1000 | 4,157 | 9.64 |
| 1000-1200 | 3,271 | 7.59 |
| 1200-1400 | 2,798 | 6.49 |
| 1400-1600 | 2,365 | 5.49 |
| 1600-1800 | 2,003 | 4.65 |
| 1800-2000 | 1,65 | 3.83 |
| 2000-2500 | 2,969 | 6.89 |
| 2500-3000 | 1,911 | 4.43 |
| 3000-4000 | 1,83 | 4.25 |
| >4000 | 1,443 | 3.35 |
| Total | 43,101 | 100.00 |

Supplementary Table S2. Distribution of isoforms per ‘gene’ in the gold standard transcriptome assembly.

| **Number of Isoforms** | **Number of Hits** | **Percentage of Hits (%)** |
| --- | --- | --- |
| 1 | 20,285 | 70.83 |
| 2 | 5,254 | 18.35 |
| 3 | 1,696 | 5.92 |
| 4 | 736 | 2.57 |
| 5 | 299 | 1.04 |
| 6 | 161 | 0.56 |
| 7 | 74 | 0.26 |
| 8 | 60 | 0.21 |
| 9 | 22 | 0.08 |
| 10 | 17 | 0.06 |
| 11 | 8 | 0.03 |
| >12 | 27 | 0.09 |
| Total | 28,639 | 100.00 |

Supplementary Table S3. BUSCO evaluations of completeness of C. macropomum Liver gold standard transcriptome against the metazoa and eukaryota gene sets.

| **BUSCO Statistics** | **Metazoa Database (%)** | **Eukaryota Database (%)** |
| --- | --- | --- |
| **Complete** | 84.7 | 82.2 |
| **Single** | 69.6 | 69.3 |
| **Multi** | 15.1 | 12.9 |
| **Fragment** | 8.5 | 10.9 |
| **Missing** | 6.8 | 6.9 |
| **Total Busco Groups** | 978 | 303 |

Supplementary Table S4. The top 20 species for which there was a top Blast-x hit. These Blast-x results from the queried of gold standard transcriptome against Non-redundant Database of NCBI.

| **Specie** | **Taxon ID** | **Number of Blast-x Hits** | **Percentage of Blast-x Hits (%)** |
| --- | --- | --- | --- |
| *Pygocentrus nattereri* | 42514 | 33,232 | 78.66 |
| *Astyanax mexicanus* | 7994 | 2,521 | 5.97 |
| *Cyprinus carpio* | 7962 | 778 | 1.84 |
| *Ictalurus punctatus* | 7998 | 744 | 1.76 |
| *Oncorhynchus mykiss* | 8022 | 669 | 1.58 |
| *Danio rerio* | 7955 | 562 | 1.33 |
| *Larimichthys crocea* | 215358 | 498 | 1.18 |
| *Sinocyclocheilus anshuiensis* | 1608454 | 270 | 0.64 |
| *Sinocyclocheilus grahami* | 75366 | 205 | 0.49 |
| *Sinocyclocheilus rhinocerous* | 307959 | 192 | 0.45 |
| *Scleropages formosus* | 113540 | 177 | 0.42 |
| *Oreochromis niloticus* | 8128 | 171 | 0.40 |
| *Salmo salar* | 8030 | 154 | 0.36 |
| *Austrofundulus limnaeus* | 52670 | 141 | 0.33 |
| *Clupea harengus* | 7950 | 138 | 0.33 |
| *Oryzias latipes* | 8090 | 133 | 0.31 |
| *Esox lucius* | 8010 | 78 | 0.18 |
| *Notothenia coriiceps* | 8208 | 78 | 0.18 |
| *Lepisosteus oculatus* | 7918 | 77 | 0.18 |
| *Maylandia zebra* | 106582 | 72 | 0.17 |
| *Others* | - | 1,356 | 6.4 |
| Total | - | 42,246 | 42.246 |

Supplementary Table S5**.** E-value distribution of Blast-x hits of gold standard transcriptome against NR database.

| **E-values Ranges** | **Number of Blast-x Hits** | **Percentage of Blast-x Hits (%)** |
| --- | --- | --- |
| 0 ~ 1e-100 | 18,213 | 43.1 |
| 1e-100 ~ 1e-60 | 8,954 | 21.2 |
| 1e-60 ~ 1e-45 | 5,256 | 12.4 |
| 1e-45 ~ 1e-30 | 4,400 | 10.4 |
| 1e-30 ~ 1e-15 | 3,414 | 8.1 |
| 1e-15 ~ 1e-5 | 2,009 | 4.8 |
| Total | 42,246 | 100.00 |

Supplementary Table S6. Similarity distribution of Blast-x hits of gold standard transcriptome against NR database.

| **Similarity Ranges (%)** | **Number of Blast-x Hits** | **Percentage of Blast-x Hits (%)** |
| --- | --- | --- |
| 20 ~ 40 | 34 | 0.1 |
| 40 ~ 60 | 1,109 | 2.6 |
| 60 ~ 80 | 4,271 | 10.1 |
| 80 ~ 90 | 5,812 | 13.8 |
| 90 ~ 95 | 6,842 | 16.2 |
| 95 ~ 100 | 24,178 | 57.2 |
| Total | 42,246 | 100.00 |

Supplementary Table S7. Functional annotation categories and statistics for gold standard transcriptome and for unigenes.

| **Trinotate Annotation Statistics** | **Final transcriptome assembly** | **Final Transcriptome Subset** |
| --- | --- | --- |
| Number of “genes” with ORF | 28,639 | - |
| Number of “Unigenes” with ORF | - | 28,639 |
| Number of transcripts with ORF | 43,101 | 28,639 |
| Transcripts with Blastx match NR | 42,246 | 28,067 |
| Transcripts with Blastp match NR | 40,046 | 26,555 |
| Transcripts with Blastx match Uniref90 | 42,295 | 28,099 |
| Transcripts with Blastp match Uniref90 | 40,187 | 26,648 |
| Transcripts with Blastx match Trembl | 40,981 | 27,296 |
| Transcripts with Blastp match Trembl | 39,256 | 25,958 |
| Transcripts with Blastx match SwissProt | 34,426 | 22,423 |
| Transcripts with Blastp match SwissProt | 33,135 | 21,642 |
| Transcripts with GO terms | 32,334 | 21,075 |
| Transcripts with eggNOG/COG | 28,483 | 18,750 |
| Transcripts with PFAM | 28,301 | 18,297 |

Supplementary Table S8. Clusters of orthologous groups distribution.

| **Class** | **Number of Unigenes** | **Percentage of Unigenes (%)** | **Class Description** | |  |
| --- | --- | --- | --- | --- | --- |
| **A** | 50 | 0.80 | RNA processing and modification | |  |
| **B** | 159 | 2.56 | Chromatin structure and dynamics | |  |
| **C** | 171 | 2.75 | Energy production and conversion | |  |
| **D** | 92 | 1.48 | Cell cycle control, cell division, chromosome partitioning | |  |
| **E** | 267 | 4.29 | Amino acid transport and metabolism | |  |
| **F** | 135 | 2.17 | Nucleotide transport and metabolism | |  |
| **G** | 218 | 3.51 | Carbohydrate transport and metabolism | |  |
| **H** | 113 | 1.82 | Coenzyme transport and metabolism | |  |
| **I** | 236 | 3.79 | Lipid transport and metabolism | |  |
| **J** | 339 | 5.45 | Translation, ribosomal structure and biogenesis | |  |
| **K** | 281 | 4.52 | Transcription | |  |
| **L** | 284 | 4.57 | Replication, recombination and repair | |  |
| **M** | 64 | 1.03 | Cell wall/membrane/envelope biogenesis | |  |
| **N** | 4 | 0.06 | Cell motility | |  |
| **O** | 650 | 10.45 | Posttranslational modification, protein turnover, chaperones | |  |
| **P** | 184 | 2.96 | Inorganic ion transport and metabolism | |  |
| **Q** | 180 | 2.89 | Secondary metabolites biosynthesis, transport and catabolism | |  |
| **R** | 1,509 | 0.02 | General function prediction only | |  |
| **S** | 268 | 4.31 | Function unknown | |  |
| **T** | 386 | 6.21 | Signal transduction mechanisms | |  |
| **U** | 184 | 2.96 | Intracellular trafficking, secretion, and vesicular transport | |  |
| **V** | 97 | 1.56 | Defence mechanisms | |  |
| **W** | 0 | 0.00 | Extracellular structures | |  |
| **Y** | 10 | 0.16 | Nuclear structure | |  |
| **Z** | 338 | 5.43 | Cytoskeleton | |  |
| **Total** | 6,219 | 100.00 |  |  | |

Supplementary Table S9. Total number of KEGG pathways with the numbers of transcripts of tambaqui.

| **ID** | **Pathway** | **No. of sequences** |
| --- | --- | --- |
| 00010 | Glycolysis / Gluconeogenesis | 33 |
| 00020 | Citrate cycle (TCA cycle) | 23 |
| 00030 | Pentose phosphate pathway | 18 |
| 00040 | Pentose and glucuronate interconversions | 16 |
| 00051 | Fructose and mannose metabolism | 21 |
| 00052 | Galactose metabolism | 17 |
| 00053 | Ascorbate and aldarate metabolism | 8 |
| 00061 | Fatty acid biosynthesis | 7 |
| 00062 | Fatty acid elongation | 17 |
| 00071 | Fatty acid degradation | 25 |
| 00072 | Synthesis and degradation of ketone bodies | 5 |
| 00073 | Cutin, suberine and wax biosynthesis | 1 |
| 00100 | Steroid biosynthesis | 16 |
| 00120 | Primary bile acid biosynthesis | 12 |
| 00130 | Ubiquinone and other terpenoid-quinone biosynthesis | 10 |
| 00140 | Steroid hormone biosynthesis | 18 |
| 00190 | Oxidative phosphorylation | 81 |
| 00220 | Arginine biosynthesis | 12 |
| 00230 | Purine metabolism | 108 |
| 00232 | Caffeine metabolism | 3 |
| 00240 | Pyrimidine metabolism | 65 |
| 00250 | Alanine, aspartate and glutamate metabolism | 23 |
| 00254 | Aflatoxin biosynthesis | 1 |
| 00260 | Glycine, serine and threonine metabolism | 31 |
| 00261 | Monobactam biosynthesis | 1 |
| 00270 | Cysteine and methionine metabolism | 31 |
| 00280 | Valine, leucine and isoleucine degradation | 40 |
| 00281 | Geraniol degradation | 3 |
| 00290 | Valine, leucine and isoleucine biosynthesis | 2 |
| 00300 | Lysine biosynthesis | 1 |
| 00310 | Lysine degradation | 46 |
| 00311 | Penicillin and cephalosporin biosynthesis | 1 |
| 00330 | Arginine and proline metabolism | 27 |
| 00333 | Prodigiosin biosyntheses | 1 |
| 00340 | Histidine metabolism | 13 |
| 00350 | Tyrosine metabolism | 16 |
| 00360 | Phenylalanine metabolism | 11 |
| 00361 | Chlorocyclohexane and chlorobenzene degradation | 1 |
| 00362 | Benzoate degradation | 2 |
| 00364 | Fluorobenzoate degradation | 1 |
| 00380 | Tryptophan metabolism | 26 |
| 00400 | Phenylalanine, tyrosine and tryptophan biosynthesis | 4 |
| 00401 | Novobiocin biosynthesis | 1 |
| 00410 | beta-Alanine metabolism | 20 |
| 00430 | Taurine and hypotaurine metabolism | 5 |
| 00440 | Phosphonate and phosphinate metabolism | 4 |
| 00450 | Selenocompound metabolism | 10 |
| 00460 | Cyanoamino acid metabolism | 4 |
| 00471 | D-Glutamine and D-glutamate metabolism | 3 |
| 00472 | D-Arginine and D-ornithine metabolism | 1 |
| 00480 | Glutathione metabolism | 24 |
| 00500 | Starch and sucrose metabolism | 19 |
| 00510 | N-Glycan biosynthesis | 35 |
| 00511 | Other glycan degradation | 14 |
| 00512 | Mucin type O-glycan biosynthesis | 5 |
| 00513 | Various types of N-glycan biosynthesis | 28 |
| 00514 | Other types of O-glycan biosynthesis | 15 |
| 00515 | Mannose type O-glycan biosyntheis | 17 |
| 00520 | Amino sugar and nucleotide sugar metabolism | 36 |
| 00521 | Streptomycin biosynthesis | 6 |
| 00523 | Polyketide sugar unit biosynthesis | 1 |
| 00524 | Neomycin, kanamycin and gentamicin biosynthesis | 2 |
| 00525 | Acarbose and validamycin biosynthesis | 1 |
| 00531 | Glycosaminoglycan degradation | 11 |
| 00532 | Glycosaminoglycan biosynthesis - chondroitin sulfate / dermatan sulfate | 15 |
| 00533 | Glycosaminoglycan biosynthesis - keratan sulfate | 10 |
| 00534 | Glycosaminoglycan biosynthesis - heparan sulfate / heparin | 17 |
| 00561 | Glycerolipid metabolism | 28 |
| 00562 | Inositol phosphate metabolism | 45 |
| 00563 | Glycosylphosphatidylinositol(GPI)-anchor biosynthesis | 18 |
| 00564 | Glycerophospholipid metabolism | 49 |
| 00565 | Ether lipid metabolism | 20 |
| 00590 | Arachidonic acid metabolism | 19 |
| 00591 | Linoleic acid metabolism | 6 |
| 00592 | alpha-Linolenic acid metabolism | 7 |
| 00600 | Sphingolipid metabolism | 29 |
| 00601 | Glycosphingolipid biosynthesis - lacto and neolacto series | 10 |
| 00603 | Glycosphingolipid biosynthesis - globo and isoglobo series | 5 |
| 00604 | Glycosphingolipid biosynthesis - ganglio series | 8 |
| 00620 | Pyruvate metabolism | 25 |
| 00623 | Toluene degradation | 1 |
| 00625 | Chloroalkane and chloroalkene degradation | 3 |
| 00626 | Naphthalene degradation | 1 |
| 00627 | Aminobenzoate degradation | 4 |
| 00630 | Glyoxylate and dicarboxylate metabolism | 23 |
| 00633 | Nitrotoluene degradation | 1 |
| 00640 | Propanoate metabolism | 24 |
| 00643 | Styrene degradation | 3 |
| 00650 | Butanoate metabolism | 15 |
| 00660 | C5-Branched dibasic acid metabolism | 1 |
| 00670 | One carbon pool by folate | 14 |
| 00680 | Methane metabolism | 14 |
| 00710 | Carbon fixation in photosynthetic organisms | 15 |
| 00720 | Carbon fixation pathways in prokaryotes | 10 |
| 00730 | Thiamine metabolism | 6 |
| 00740 | Riboflavin metabolism | 6 |
| 00750 | Vitamin B6 metabolism | 6 |
| 00760 | Nicotinate and nicotinamide metabolism | 16 |
| 00770 | Pantothenate and CoA biosynthesis | 10 |
| 00780 | Biotin metabolism | 3 |
| 00785 | Lipoic acid metabolism | 3 |
| 00790 | Folate biosynthesis | 16 |
| 00830 | Retinol metabolism | 19 |
| 00860 | Porphyrin and chlorophyll metabolism | 22 |
| 00900 | Terpenoid backbone biosynthesis | 16 |
| 00901 | Indole alkaloid biosynthesis | 1 |
| 00903 | Limonene and pinene degradation | 1 |
| 00908 | Zeatin biosynthesis | 1 |
| 00909 | Sesquiterpenoid and triterpenoid biosynthesis | 1 |
| 00910 | Nitrogen metabolism | 4 |
| 00920 | Sulfur metabolism | 8 |
| 00930 | Caprolactam degradation | 6 |
| 00940 | Phenylpropanoid biosynthesis | 2 |
| 00944 | Flavone and flavonol biosynthesis | 1 |
| 00950 | Isoquinoline alkaloid biosynthesis | 6 |
| 00960 | Tropane, piperidine and pyridine alkaloid biosynthesis | 4 |
| 00965 | Betalain biosynthesis | 2 |
| 00970 | Aminoacyl-tRNA biosynthesis | 27 |
| 00980 | Metabolism of xenobiotics by cytochrome P450 | 11 |
| 00981 | Insect hormone biosynthesis | 2 |
| 00982 | Drug metabolism - cytochrome P450 | 8 |
| 00983 | Drug metabolism - other enzymes | 24 |
| 01040 | Biosynthesis of unsaturated fatty acids | 14 |
| 01051 | Biosynthesis of ansamycins | 1 |
| 01055 | Biosynthesis of vancomycin group antibiotics | 1 |
| 01521 | EGFR tyrosine kinase inhibitor resistance | 52 |
| 01522 | Endocrine resistance | 55 |
| 01523 | Antifolate resistance | 24 |
| 01524 | Platinum drug resistance | 39 |
| 02010 | ABC transporters | 27 |
| 02020 | Two-component system | 9 |
| 02024 | Quorum sensing | 3 |
| 02026 | Biofilm formation - Escherichia coli | 1 |
| 03008 | Ribosome biogenesis in eukaryotes | 61 |
| 03010 | Ribosome | 104 |
| 03013 | RNA transport | 118 |
| 03015 | mRNA surveillance pathway | 54 |
| 03018 | RNA degradation | 54 |
| 03020 | RNA polymerase | 25 |
| 03022 | Basal transcription factors | 29 |
| 03030 | DNA replication | 26 |
| 03040 | Spliceosome | 100 |
| 03050 | Proteasome | 41 |
| 03060 | Protein export | 18 |
| 03070 | Bacterial secretion system | 2 |
| 03320 | PPAR signaling pathway | 40 |
| 03410 | Base excision repair | 25 |
| 03420 | Nucleotide excision repair | 33 |
| 03430 | Mismatch repair | 17 |
| 03440 | Homologous recombination | 23 |
| 03450 | Non-homologous end-joining | 12 |
| 03460 | Fanconi anemia pathway | 31 |
| 04010 | MAPK signaling pathway | 171 |
| 04011 | MAPK signaling pathway - yeast | 17 |
| 04012 | ErbB signaling pathway | 49 |
| 04013 | MAPK signaling pathway - fly | 49 |
| 04014 | Ras signaling pathway | 121 |
| 04015 | Rap1 signaling pathway | 119 |
| 04016 | MAPK signaling pathway - plant | 5 |
| 04020 | Calcium signaling pathway | 79 |
| 04022 | cGMP - PKG signaling pathway | 76 |
| 04024 | cAMP signaling pathway | 91 |
| 04060 | Cytokine-cytokine receptor interaction | 108 |
| 04062 | Chemokine signaling pathway | 81 |
| 04064 | NF-kappa B signaling pathway | 68 |
| 04066 | HIF-1 signaling pathway | 57 |
| 04068 | FoxO signaling pathway | 80 |
| 04070 | Phosphatidylinositol signaling system | 49 |
| 04071 | Sphingolipid signaling pathway | 61 |
| 04072 | Phospholipase D signaling pathway | 74 |
| 04080 | Neuroactive ligand-receptor interaction | 92 |
| 04110 | Cell cycle | 73 |
| 04111 | Cell cycle - yeast | 39 |
| 04112 | Cell cycle - Caulobacter | 3 |
| 04113 | Meiosis - yeast | 29 |
| 04114 | Oocyte meiosis | 46 |
| 04115 | p53 signaling pathway | 41 |
| 04120 | Ubiquitin mediated proteolysis | 101 |
| 04122 | Sulfur relay system | 6 |
| 04130 | SNARE interactions in vesicular transport | 26 |
| 04136 | Autophagy - other eukaryotes | 22 |
| 04137 | Mitophagy - animal | 49 |
| 04138 | Autophagy - yeast | 49 |
| 04139 | Mitophagy - yeast | 17 |
| 04140 | Autophagy - animal | 91 |
| 04141 | Protein processing in endoplasmic reticulum | 115 |
| 04142 | Lysosome | 93 |
| 04144 | Endocytosis | 144 |
| 04145 | Phagosome | 74 |
| 04146 | Peroxisome | 66 |
| 04150 | mTOR signaling pathway | 92 |
| 04151 | PI3K-Akt signaling pathway | 172 |
| 04152 | AMPK signaling pathway | 68 |
| 04210 | Apoptosis | 89 |
| 04211 | Longevity regulating pathway - mammal | 56 |
| 04212 | Longevity regulating pathway - worm | 41 |
| 04213 | Longevity regulating pathway - multiple species | 33 |
| 04214 | Apoptosis - fly | 38 |
| 04215 | Apoptosis - multiple species | 19 |
| 04216 | Ferroptosis | 27 |
| 04217 | Necroptosis | 77 |
| 04218 | Cellular senescence | 90 |
| 04260 | Cardiac muscle contraction | 33 |
| 04261 | Adrenergic signaling in cardiomyocytes | 56 |
| 04270 | Vascular smooth muscle contraction | 61 |
| 04310 | Wnt signaling pathway | 66 |
| 04320 | Dorso-ventral axis formation | 13 |
| 04330 | Notch signaling pathway | 26 |
| 04340 | Hedgehog signaling pathway | 28 |
| 04341 | Hedgehog signaling pathway - fly | 17 |
| 04350 | TGF-beta signaling pathway | 53 |
| 04360 | Axon guidance | 84 |
| 04370 | VEGF signaling pathway | 31 |
| 04371 | Apelin signaling pathway | 64 |
| 04380 | Osteoclast differentiation | 77 |
| 04390 | Hippo signaling pathway | 76 |
| 04391 | Hippo signaling pathway #NAME? | 35 |
| 04392 | Hippo signaling pathway - multiple species | 15 |
| 04510 | Focal adhesion | 108 |
| 04512 | ECM-receptor interaction | 39 |
| 04514 | Cell adhesion molecules (CAMs) | 65 |
| 04520 | Adherens junction | 52 |
| 04530 | Tight junction | 90 |
| 04540 | Gap junction | 42 |
| 04550 | Signaling pathways regulating pluripotency of stem cells | 64 |
| 04610 | Complement and coagulation cascades | 58 |
| 04611 | Platelet activation | 68 |
| 04612 | Antigen processing and presentation | 32 |
| 04614 | Renin-angiotensin system | 9 |
| 04620 | Toll-like receptor signaling pathway | 56 |
| 04621 | NOD-like receptor signaling pathway | 95 |
| 04622 | RIG-I-like receptor signaling pathway | 42 |
| 04623 | Cytosolic DNA-sensing pathway | 33 |
| 04624 | Toll and Imd signaling pathway | 24 |
| 04626 | Plant-pathogen interaction | 6 |
| 04630 | Jak-STAT signaling pathway | 74 |
| 04640 | Hematopoietic cell lineage | 43 |
| 04650 | Natural killer cell mediated cytotoxicity | 48 |
| 04657 | IL-17 signaling pathway | 50 |
| 04658 | Th1 and Th2 cell differentiation | 47 |
| 04659 | Th17 cell differentiation | 59 |
| 04660 | T cell receptor signaling pathway | 56 |
| 04662 | B cell receptor signaling pathway | 45 |
| 04664 | Fc epsilon RI signaling pathway | 32 |
| 04666 | Fc gamma R-mediated phagocytosis | 51 |
| 04668 | TNF signaling pathway | 66 |
| 04670 | Leukocyte transendothelial migration | 62 |
| 04672 | Intestinal immune network for IgA production | 16 |
| 04710 | Circadian rhythm | 23 |
| 04711 | Circadian rhythm - fly | 5 |
| 04712 | Circadian rhythm - plant | 3 |
| 04713 | Circadian entrainment | 37 |
| 04720 | Long-term potentiation | 31 |
| 04721 | Synaptic vesicle cycle | 32 |
| 04722 | Neurotrophin signaling pathway | 74 |
| 04723 | Retrograde endocannabinoid signaling | 63 |
| 04724 | Glutamatergic synapse | 41 |
| 04725 | Cholinergic synapse | 42 |
| 04726 | Serotonergic synapse | 34 |
| 04727 | GABAergic synapse | 24 |
| 04728 | Dopaminergic synapse | 47 |
| 04730 | Long-term depression | 30 |
| 04740 | Olfactory transduction | 14 |
| 04742 | Taste transduction | 15 |
| 04744 | Phototransduction | 4 |
| 04745 | Phototransduction - fly | 10 |
| 04750 | Inflammatory mediator regulation of TRP channels | 40 |
| 04810 | Regulation of actin cytoskeleton | 109 |
| 04910 | Insulin signaling pathway | 72 |
| 04911 | Insulin secretion | 33 |
| 04912 | GnRH signaling pathway | 45 |
| 04913 | Ovarian Steroidogenesis | 18 |
| 04914 | Progesterone-mediated oocyte maturation | 39 |
| 04915 | Estrogen signaling pathway | 46 |
| 04916 | Melanogenesis | 36 |
| 04917 | Prolactin signaling pathway | 39 |
| 04918 | Thyroid hormone synthesis | 32 |
| 04919 | Thyroid hormone signaling pathway | 72 |
| 04920 | Adipocytokine signaling pathway | 39 |
| 04921 | Oxytocin signaling pathway | 69 |
| 04922 | Glucagon signaling pathway | 50 |
| 04923 | Regulation of lipolysis in adipocyte | 28 |
| 04924 | Renin secretion | 30 |
| 04925 | Aldosterone synthesis and secretion | 41 |
| 04926 | Relaxin signaling pathway | 50 |
| 04927 | Cortisol synthesis and secretion | 27 |
| 04930 | Type II diabetes mellitus | 22 |
| 04931 | Insulin resistance | 58 |
| 04932 | Non-alcoholic fatty liver disease (NAFLD) | 104 |
| 04933 | AGE-RAGE signaling pathway in diabetic complications | 57 |
| 04940 | Type I diabetes mellitus | 16 |
| 04950 | Maturity onset diabetes of the young | 11 |
| 04960 | Aldosterone-regulated sodium reabsorption | 16 |
| 04961 | Endocrine and other factor-regulated calcium reabsorption | 24 |
| 04962 | Vasopressin-regulated water reabsorption | 26 |
| 04964 | Proximal tubule bicarbonate reclamation | 11 |
| 04966 | Collecting duct acid secretion | 14 |
| 04970 | Salivary secretion | 32 |
| 04971 | Gastric acid secretion | 25 |
| 04972 | Pancreatic secretion | 45 |
| 04973 | Carbohydrate digestion and absorption | 14 |
| 04974 | Protein digestion and absorption | 31 |
| 04975 | Fat digestion and absorption | 20 |
| 04976 | Bile secretion | 35 |
| 04977 | Vitamin digestion and absorption | 15 |
| 04978 | Mineral absorption | 15 |
| 04979 | Cholesterol metabolism | 39 |
| 05010 | Alzheimer's disease | 114 |
| 05012 | Parkinson's disease | 87 |
| 05014 | Amyotrophic lateral sclerosis (ALS) | 31 |
| 05016 | Huntington's disease | 119 |
| 05020 | Prion diseases | 23 |
| 05030 | Cocaine addiction | 22 |
| 05031 | Amphetamine addiction | 28 |
| 05032 | Morphine addiction | 27 |
| 05033 | Nicotine addiction | 8 |
| 05034 | Alcoholism | 50 |
| 05100 | Bacterial invasion of epithelial cells | 46 |
| 05110 | Vibrio cholerae infection | 30 |
| 05120 | Epithelial cell signaling in Helicobacter pylori infection | 47 |
| 05130 | Pathogenic Escherichia coli infection | 26 |
| 05131 | Shigellosis | 40 |
| 05132 | Salmonella infection | 41 |
| 05133 | Pertussis | 43 |
| 05134 | Legionellosis | 36 |
| 05140 | Leishmaniasis | 43 |
| 05142 | Chagas disease (American trypanosomiasis) | 61 |
| 05143 | African trypanosomiasis | 18 |
| 05144 | Malaria | 26 |
| 05145 | Toxoplasmosis | 62 |
| 05146 | Amoebiasis | 49 |
| 05150 | Staphylococcus aureus infection | 25 |
| 05152 | Tuberculosis | 87 |
| 05160 | Hepatitis C | 67 |
| 05161 | Hepatitis B | 87 |
| 05162 | Measles | 72 |
| 05164 | Influenza A | 91 |
| 05165 | Human papillomavirus infection | 166 |
| 05166 | HTLV-I infection | 146 |
| 05167 | Kaposi's sarcoma-associated herpesvirus infection | 97 |
| 05168 | Herpes simplex infection | 109 |
| 05169 | Epstein-Barr virus infection | 130 |
| 05200 | Pathways in cancer | 285 |
| 05202 | Transcriptional misregulation in cancers | 110 |
| 05203 | Viral carcinogenesis | 112 |
| 05204 | Chemical carcinogenesis | 13 |
| 05205 | Proteoglycans in cancer | 124 |
| 05206 | MicroRNAs in cancer | 99 |
| 05210 | Colorectal cancer | 53 |
| 05211 | Renal cell carcinoma | 48 |
| 05212 | Pancreatic cancer | 51 |
| 05213 | Endometrial cancer | 38 |
| 05214 | Glioma | 39 |
| 05215 | Prostate cancer | 61 |
| 05216 | Thyroid cancer | 26 |
| 05217 | Basal cell carcinoma | 28 |
| 05218 | Melanoma | 37 |
| 05219 | Bladder cancer | 27 |
| 05220 | Chronic myeloid leukemia | 51 |
| 05221 | Acute myeloid leukemia | 43 |
| 05222 | Small cell lung cancer | 57 |
| 05223 | Non-small cell lung cancer | 41 |
| 05224 | Breast cancer | 71 |
| 05225 | Hepatocellular carcinoma | 83 |
| 05226 | Gastric cancer | 70 |
| 05230 | Central carbon metabolism in cancer | 36 |
| 05231 | Choline metabolism in cancer | 52 |
| 05310 | Asthma | 6 |
| 05320 | Autoimmune thyroid disease | 12 |
| 05321 | Inflammatiory bowel disease (IBD) | 31 |
| 05322 | Systemic lupus erythematosus | 36 |
| 05323 | Rheumatoid arthritis | 45 |
| 05330 | Allograft rejection | 14 |
| 05332 | Graft-versus-host disease | 12 |
| 05340 | Primary immunodeficiency | 21 |
| 05410 | Hypertrophic cardiomyopathy (HCM) | 43 |
| 05412 | Arrhythmogenic right ventricular cardiomyopathy (ARVC) | 38 |
| 05414 | Dilated cardiomyopathy (DCM) | 47 |
| 05416 | Viral myocarditis | 28 |
| 05418 | Fluid shear stress and atherosclerosis | 79 |
