# Supplementary material for: From the Amazon: A comprehensive liver transcriptome dataset of the teleost fish tambaqui, Colossoma macropomum
Source: Data Brief. 2019 Mar 7;23:103751. doi: 10.1016/j.dib.2019.103751 (PMC6660486; doi:10.1016/j.dib.2019.103751)
Supplement: Multimedia component 1 [file mmc1.pdf]

ciimar
Centro Interdisciplinar
de Investigação
Marinha e Ambiental

**Filipe Castro**, PhD Biomedical Sciences
CIIMAR – Interdisciplinary Centre of Marine and Environmental Research, U. Porto – University of Porto, Porto, Portugal
Department of Biology, Faculty of Sciences, U. Porto - University of Porto, Portugal
http://castroevolab.com

Dear Editor,

Please note that all authors have read and approved the manuscript for submission and that the authors have no competing interests to declare. The content of the manuscript has not been published or submitted for publication elsewhere.

Sincerely Yours

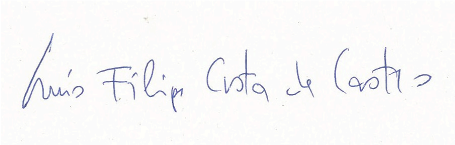

Luís Filipe Costa Castro
(on behalf of all authors)

Terminal de Cruzeiros do Porto de Leixões
Avenida General Norton de Matos, S/N
4450-208 Matosinhos | Portugal

Tel.: (351) 22 340 18 00
Fax: (351) 22 339 06 08

www.ciimar.up.pt
